# Azole Resistance Mechanisms in Pathogenic Malassezia furfur

**DOI:** 10.1128/AAC.01975-20

**Published:** 2021-04-19

**Authors:** Cheryl Leong, Joel Wai Kit Chan, Shi Mun Lee, Yuen In Lam, Joleen P. Z. Goh, Giuseppe Ianiri, Thomas L. Dawson

**Affiliations:** a Skin Research Institute of Singapore, Agency for Science, Technology and Research, Singapore, Singapore; b Department of Agricultural, Environmental and Food Sciences, University of Molise, Campobasso, Italy; c Center for Cell Death, Injury and Regeneration, Department of Drug Discovery and Biomedical Sciences, College of Pharmacy, Medical University of South Carolina, Charleston, South Carolina, USA

**Keywords:** intrinsic, acquired, resistance, azoles, *Malassezia furfur*, CYP51, PDR10, AMR, *Malassezia*, antifungal resistance, mechanisms of resistance

## Abstract

*Malassezia* spp. are emerging fungal pathogens causing opportunistic skin and severe systemic infection. Nosocomial outbreaks are associated with azole resistance, and understanding of the underlying mechanisms is limited to knowledge of other fungal species. Here, we identified distinct antifungal susceptibility patterns in 26 Malassezia furfur isolates derived from healthy and diseased individuals. A Y67F *CYP51* mutation was identified in five isolates of *M. furfur*. However, this mutation alone was insufficient to induce reductions in azole susceptibility in the wild-type strain. RNA sequencing (RNA-seq) and differential gene analysis of strains derived from individuals with healthy or disease backgrounds exposed to clotrimazole *in vitro* identified several key metabolic pathways and transporter proteins which are involved in reducing azole susceptibility. The pleiotropic drug transporter gene *PDR10* was the single most highly upregulated transporter gene in multiple strains of *M. furfur* after azole treatment, and increased expression of *PDR10* is associated with reduced azole susceptibility in some systemic-disease isolates of *M. furfur*. Deletion of *PDR10* in a pathogenic *M. furfur* strain with reduced susceptibility reduced MICs to those of susceptible isolates. In light of the current dearth of antifungal technologies, globally emerging multiazole resistance, and broad use of azoles in agriculture and consumer care, an improved understanding of the mechanisms underlying intrinsic and acquired azole resistance in *Malassezia* is crucial for development of antibiotic stewardship and antifungal treatment strategies.

## Abstract (precis)

*Malassezia* spp. are emerging fungal pathogens causing opportunistic skin and severe systemic infection. Nosocomial outbreaks are associated with azole resistance, and understanding of the underlying mechanisms is limited to knowledge of other fungal species.

## INTRODUCTION

*Malassezia*, the lipid-dependent skin commensal, has also been shown to be an emerging pathogen not just in neonatal intensive care wards but also in Crohn’s disease and pancreatic cancers (1–4). *Malassezia*-associated superficial mycoses (dandruff, pityriasis versicolor, and folliculitis) affect up to 50% of the global population (5) and have the potential to cause severe systemic and invasive infection in immunocompromised individuals (6–9). Outbreaks in neonatal and intensive care wards have been increasingly reported (6). Azole antifungals are the primary antifungal treatment for *Malassezia*-associated diseases due to their intrinsic resistance to some antifungals, particularly echinocandins (10). Because reduced *in vitro* antifungal susceptibility may be an indicator of clinical failure (11), there is a need to profile the susceptibility of *Malassezia* isolates derived from individuals with healthy or disease backgrounds to elucidate and design prevention strategies for the underlying mechanisms contributing to current and emerging trends of antifungal resistance.

Azole resistance may be primary (intrinsic) or secondary (acquired) (12). The former is found naturally without prior (known) antifungal exposure. The latter is a result of a previously susceptible strain being exposed to antifungals or other selective pressure and may be a result of altered gene expression, point mutations, or allelic variations (13). Both may be attributed to an increase in (i) the prophylactic use of azole drugs, (ii) prolonged treatment regimens, (iii) agricultural use of azole fungicides for crop protection (14), or (iv) the broad-spectrum, long-term, and low-dose use of azoles in consumer care. Azole treatment efficacy is variable, depending on the involved species, and resistant strains are being documented with increasing frequency (10, 15). Among human commensal *Malassezia* species, Malassezia furfur and Malassezia pachydermatis are the species most commonly encountered in systemic infection of neonates and immunocompromised persons (15). An increasing number of ketoconazole-resistant *M. pachydermatis* strains have been reported to have been found on pet dogs in Korea and can be attributed to the use of antifungal shampoo (16). The prevalence of the normally zoophilic species *M. pachydermatis* in infection is worrying evidence of animal-to-human transmission (6).

Sterol 14α-demethylase (encoded by *CYP51* [*ERG11*]) is a key cytochrome P450 enzyme involved in fungal cell wall ergosterol synthesis (17). *Malassezia* azole resistance is largely associated with mutations in the *ERG11/CYP51* gene, identified from clinical isolates (15, 18, 19). In clinical isolates derived from subjects with severe dandruff, the *CYP51* mutations Y127F, A169S, and K176 were identified in Malassezia globosa (15). Highly multiazole-resistant strains of *M. pachydermatis* have also been extensively documented and found to be associated with missense mutations in *CYP51* (20, 21). A further genomic tandem quadruplication in the *ERG5/ERG11* gene region has been described in a ketoconazole-resistant isolate of *M. pachydermatis* (18).

Fungal multidrug resistance is also associated with overexpression of transporters of the ATP-binding cassette (ABC) superfamily and major facilitator superfamily (MFS) (22–25). These transporters utilize ATP hydrolysis or electrochemical gradients to carry out efflux of antifungals, thus reducing susceptibility (22). Yeast ABC transporters are classified into six subfamilies of up to 30 unique proteins, such as *CDR1* and *MDR*, which confer drug resistance in human pathogens, including Candida albicans. In *M. furfur* and *M. pachydermatis*, the role of efflux pumps has also been implicated through the synergistic use of efflux pump modulators together with azoles to inhibit growth (26). Pleiotropic drug resistance protein 10 (encoded by *PDR10*) is involved in fluconazole resistance and has been identified in *M. furfur* (27). Genomic multiplications in *ATM1* and *ERG11*, encoding iron-sulfur transporters, have also been found to confer ketoconazole resistance in Malassezia restricta (16).

In this study, we analyzed antifungal susceptibility profiles of 26 strains of *M. furfur* derived from individuals with healthy or disease backgrounds and performed RNA sequencing (RNA-seq) to identify candidate genes driving intrinsic and acquired resistance. Although mutations in *CYP51* were originally postulated to be the main driver of elevated MICs in *M. furfur*, differential gene analysis via RNA-seq and subsequent gene validation has identified genes from the ABC transporter protein family, particularly *PDR10*, to be key drivers of intrinsic and acquired resistance in the *M. furfur* strains CBS 7982 and CBS 14141. Knowledge of the functional mechanisms underlying antifungal susceptibility in *Malassezia* will be beneficial in identifying new therapeutic targets, understanding the emergence of new resistant strains, and developing global antifungal drug stewardship strategies.

## RESULTS

### Diseased-derived isolates of *M. furfur* have elevated terbinafine, clotrimazole, and miconazole MICs.

**FIG 1:**
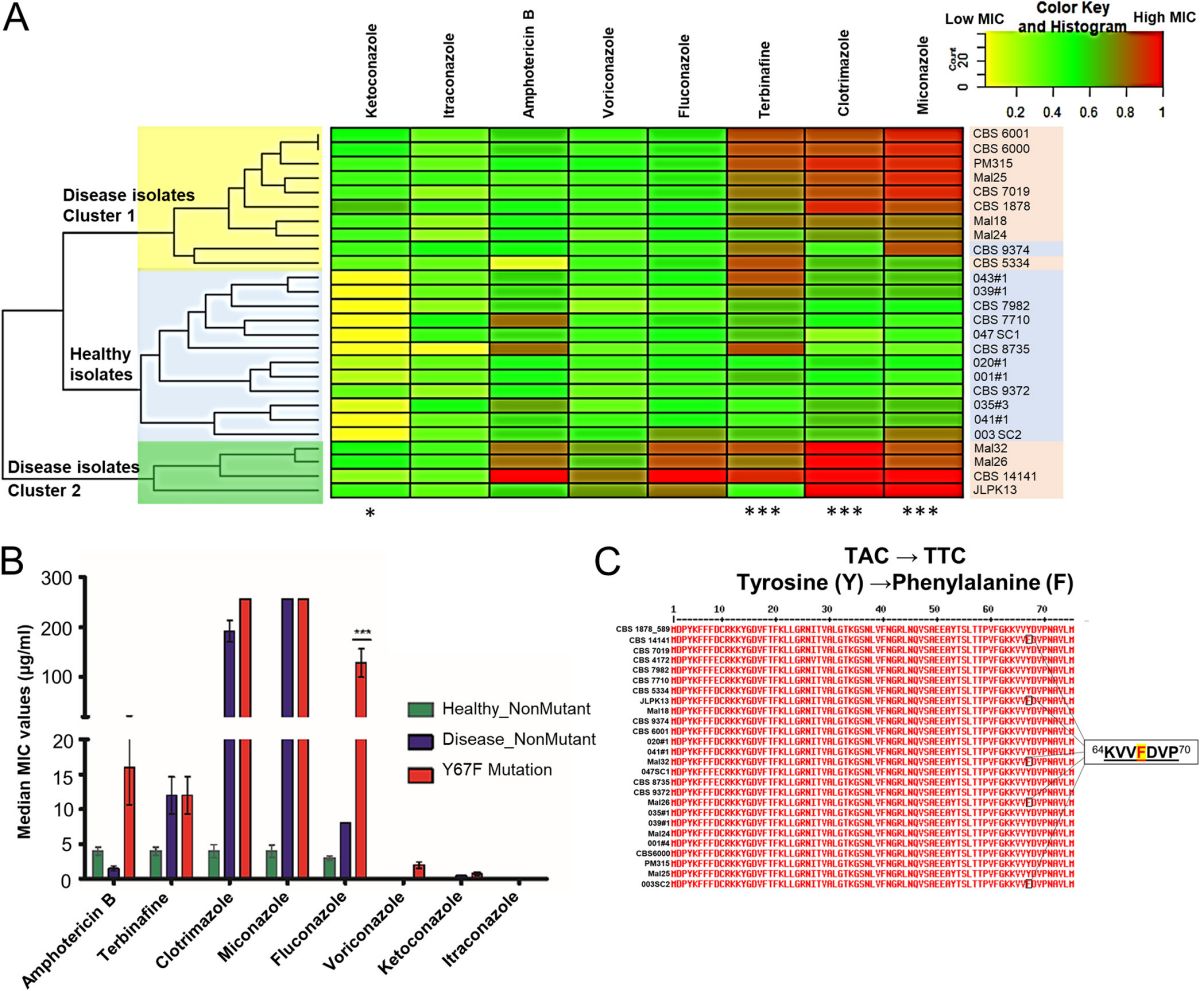
The 26 strains of *M. furfur* used, including 13 healthy and 13 disease isolates. (A) Heat map of the MICs of 8 antifungals. MICs were normalized from 0 to 1, with 1 being the highest MIC (red) for each individual compound. Strains isolated from healthy individuals are highlighted in blue, and strains from individuals with disease are highlighted in red. A paired two-tailed *t* test (*, *P* < 0.05; ***, *P* < 0.005) was performed for median MICs between healthy and disease isolates. (B) Median MICs and standard deviations (SD) for healthy and disease wild-type and Y67F mutant strains. A one-way ANOVA was used with Dunnett’s test (***, *P* < 0.005). (C) *CYP51* amino acid sequence showing the Y67F mutation in 5 isolates.

Antifungal susceptibility testing was performed on 26 strains of *M. furfur* comprising isolates from healthy human skin (referred to here as “healthy isolates”) and those from skin disease or systemic infection (“disease isolates”) (Table 1) using a panel of eight antifungals: amphotericin B (AMB), terbinafine (TRB), clotrimazole (CTZ), miconazole (MCZ), fluconazole (FLZ), voriconazole (VRZ), ketoconazole (KTZ) and itraconazole (ITZ) (Fig. 1). The MIC of each antifungal presented in Fig. 1 was normalized from 0 to 1, with 1 being the highest MIC (red) for each individual compound.

**TABLE 1:** Healthy and disease *M. furfur* isolates used in this study

Isolate	Source
Healthy-skin isolates
CBS 7982	Ear
020#01	Nose
041#01	Nose
039#01	Nose
035#03	Nose
CBS 9372	Back
CBS 9374	Chest
CBS 8735	Bronchial wash
047 SC1	Scalp
003 SC2	Scalp
CBS 7710	Skin of man
043#01	Nose
001#01	Nose
Diseased-skin isolates
CBS 5334	Infected skin
CBS 6000	Dandruff
CBS 6001	Pityriasis versicolor
CBS 1878	Dandruff
CBS 7019	Pityriasis versicolor
Systemic-disease isolates
CBS 14141	Blood
JLPK13	Urine
Mal18	Blood
Mal24	Blood
Mal25	Skin catheter insertion
Mal26	Blood
Mal32	Skin catheter insertion
PM315	Anal swab of neonate

The isolates further clustered into 2 groups, with disease isolates in cluster 2 (Fig. 1A, green) having elevated MICs for almost all azole antifungals and disease isolates in cluster 1 (Fig. 1A, yellow) having high MICs specifically for TRB, CTZ, and MCZ (*P* < 0.001) (Fig. 1B; also, see Table S1 in the supplemental material). ITZ had the lowest MICs of all antifungals tested (Fig. 1B), although disease isolates of *M. furfur* still had significantly higher MICs for KTZ (*P* < 0.05) (Fig. 1B). FLZ and VRZ MICs were higher for disease isolates in cluster 2 (Fig. 1A, green), although these values were not significant.

### *CYP51A1* Y67F mutants have elevated fluconazole and voriconazole MICs.

The gene sequence of *CYP51A1* in *M. furfur* was identified by a whole-genome-sequencing (WGS) BLAST search (BioProject ID 286710) of a known *M. globosa CYP51A1* sequence (15) against *M. furfur* genomes and validated by the identification of the presence of the highly conserved heme-binding site (FGFGRHRCIG), EXXR motif (ERLR), and conserved threonine of the I helix involved in proton delivery (Fig. S1).

A tyrosine-to-phenylalanine mutation in residue 67 (Y67F) of *CYP51A1* was identified in the blood isolate CBS 14141 (Fig. 1C; Fig. S2). The mutation was also identified in four other isolates, i.e., the systemic-disease isolates Mal24, Mal26, and JLPK13 and the healthy-skin isolate 003 SC2. The three systemic-disease isolates showed elevated MICs (Fig. 1B and C; Table S1) specifically for FLZ (*P* < 0.001), consistent with literature indicating the role of the tyrosine residue in drug binding (28). Healthy skin isolate 003 SC2 also showed slightly elevated MICs for MCZ and FLZ. This mutation is synonymous with the Y132F mutation in Candida albicans and is well known to affect binding to fluconazole due to the residue being 7 Å away from a key binding motif (29). It has been identified as a Y127F mutation in disease isolates of *M. globosa* (15).

### Y67F knock-in and F67Y rescue mutants show no changes in azole susceptibility.

**FIG 2:**
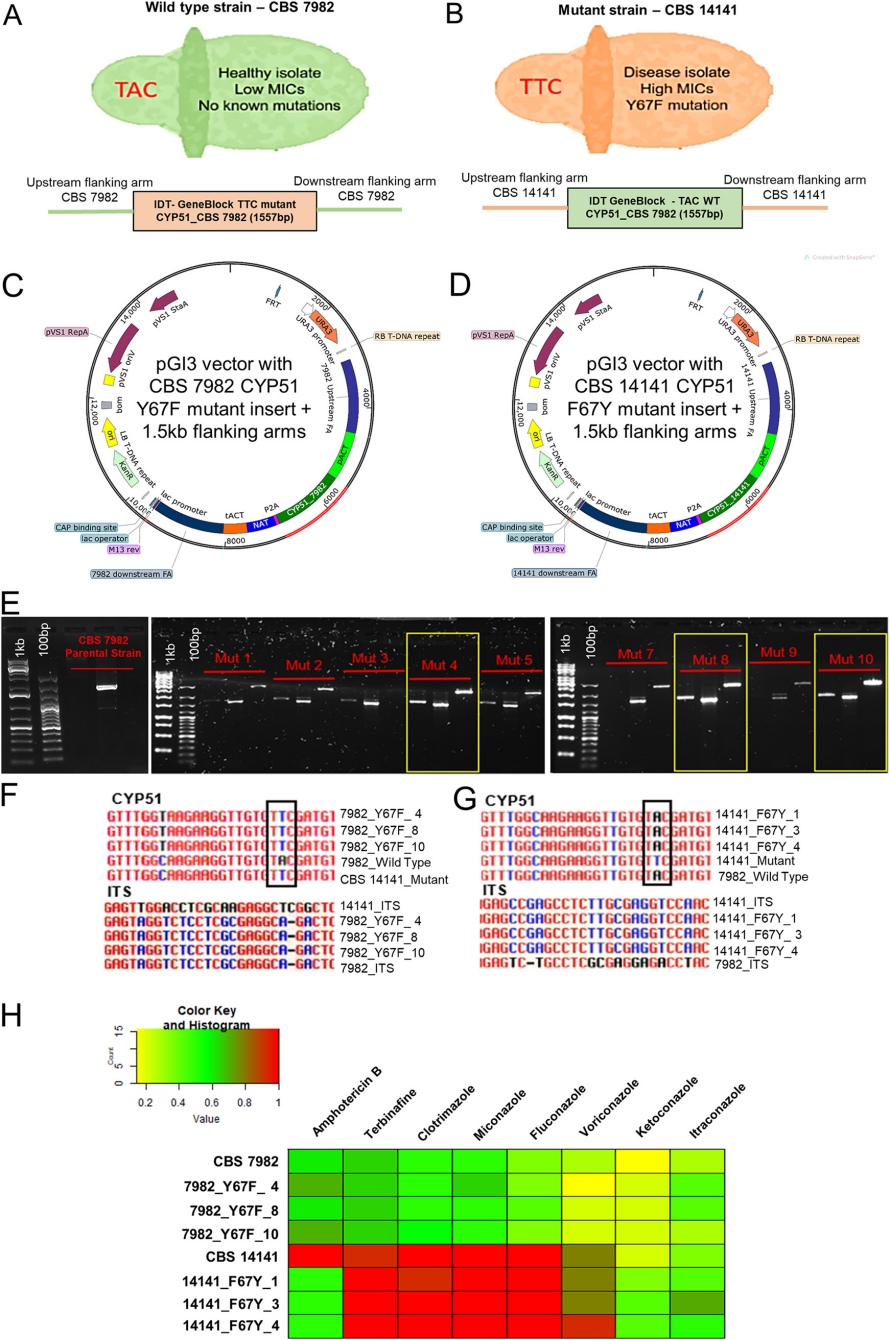
Agrobacterium tumefaciens-mediated transformation (ATMT) of *CYP51* mutation into wild-type CBS 7982 and mutant CBS 14141 *M. furfur*. (A and B) Phenotypes and genotypes of the *M. furfur* wild-type strain CBS 7982 (A) and the mutant strain CBS 14141 (B). (C and D) Vector maps for CBS 7982 (C) and CBS 14141 (D) ATMT. (E) Screening and selection of CBS 7982 transformant colonies with the correct *CYP51* insert (∼700 bp) and 1.5-kb upstream/downstream flanking arms. (F and G) Sanger sequencing validation of the correct single nucleotide mutation and ITS sequences in CBS 7982 (F) and CBS 14141 (G). (H) Heat map of the MICs for CBS 7982 Y67F and CBS 14141 F67Y mutants. MICs were normalized from 0 to 1, with 1 being the highest MIC (red).

The single *CYP51* nucleotide change coding for the Y67F mutation was inserted into the wild-type, low-MIC strain CBS 7982 via Agrobacterium tumefaciens-mediated transformation (ATMT) using a nourseothricin (NAT) expression vector (Fig. 2A to D). Similarly, a rescue F67Y *CYP51* mutation was generated in the *CYP51* mutant strain CBS 14141 (Fig. 2A to D). Colonies were screened for the presence of the correct 1.5-kb flanking arms and insert (Fig. 2E). Sanger sequencing of the *CYP51* mutation regions and internal transcribed spacer regions (ITS) was performed to validate the presence of the correct single nucleotide mutation and strain background of the respective transformants (Fig. 2F and G).

Insertion of the Y67F *CYP51* mutation into the CBS 7982 strain did not yield any change in azole susceptibility (Fig. 2H; Table 2). Similarly, an F67Y rescue mutant generated from the CBS 14141 *CYP51* mutant strain did not show any reduction in azole MICs when tested against our panel of antifungals. The F67Y rescue mutation restored amphotericin B MICs to wild-type levels in CBS 14141, although introduction into wild-type CBS 7982 increased AMB MICs only slightly.

**TABLE 2:** MICs for *CYP51* transformants

Strain	MIC (μg/ml) of^*a*^:							
	AMB	TRB	CTZ	MCZ	FLZ	VRZ	KTZ	ITZ
CBS 7982	4	2	4	4	1	0.03	0.03	0.03
Y67F_4	8	2	4	16	1	0.008	0.06	0.125
Y67F_8	4	2	4	4	2	0.015	0.06	0.125
Y67F_10	8	2	8	4	1	0.015	0.06	0.03
CBS 14141	>32	32	>256	>256	>256	4	0.06	0.06
F67Y_1	4	>32	256	>256	>256	4	0.25	0.125
F67Y_3	4	>32	>256	>256	>256	4	0.5	2
F67Y_4	4	>32	>256	>256	>256	16	0.5	0.25

a AMB, amphotericin B; TRB, terbinafine; CTZ, clotrimazole; MCZ, miconazole; FLZ, fluconazole; VRZ, voriconazole; KTZ, ketoconazole; ITZ, itraconazole.

These observations suggest that reduced azole susceptibility may be mediated by factors other than *CYP51* in *M. furfur* and that the Y67F mutation mainly impacts AMB susceptibility in our selected strains. This is supported by the observation that the healthy-skin isolate 003 SC2 contains the Y67F *CYP51* mutation but does not show the level of reduced azole susceptibility observed in disease isolate cluster 2.

### Genes involved in metabolism and secondary metabolite production are upregulated in disease strains of *M. furfur*.

To further elucidate the pathways potentially involved in *M. furfur* azole susceptibility, we performed RNA-seq in regular modified Dixon (mDixon) medium for representative candidate strains from each cluster (healthy isolates, disease isolate cluster 1, and disease isolate cluster 2) based on their antifungal susceptibility testing (AFST) profiles (Fig. 1). They were defined as the wild type, susceptible strain CBS 7982 (healthy isolate cluster), the disease *CYP51* mutant strain CBS 14141 (disease isolate cluster 2), and the intermediate disease strain CBS 7019 (disease isolate cluster 1).

**FIG 3:**
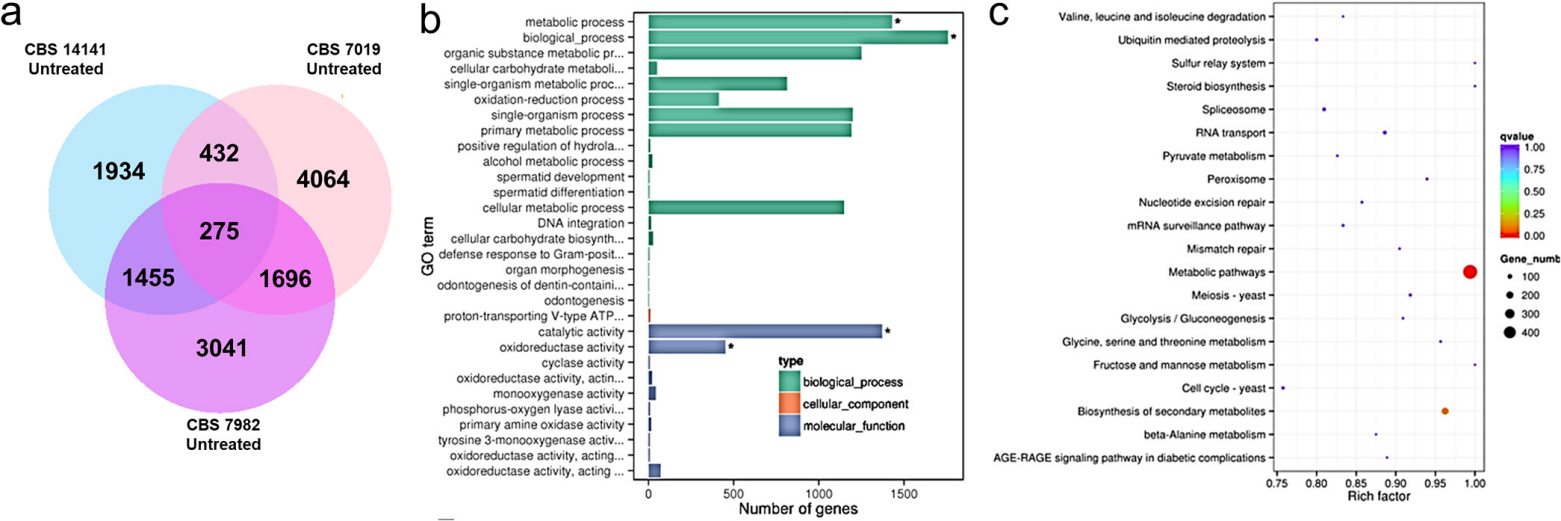
RNA-seq of CBS 7982, CBS 7019, and CBS 14141. (a) Venn diagram showing genes which are differentially expressed in CBS 7982, CBS 7019, and CBS 14141. (b) GO enrichment bar chart of differentially expressed terms. Asterisks indicate terms which are significantly enriched. (c) Scatterplot for KEGG enrichment results in CBS 7982 versus CBS 14141.

A list of the top 20 upregulated genes commonly expressed in disease isolates CBS 7019 and CBS 14141 is presented in Table S2. Genes involved in metabolism, biological process, catalytic activity, and extracellular activity were the key genes upregulated in the disease strains (Fig. 3b). Statistically enriched KEGG pathways include secondary metabolite production (Fig. 3c). Upregulation of some exons coding for ABC and MFS transporter proteins, such as *YBT1*, *ITR1*, and *OPT1*, was also observed.

### Healthy and disease isolates have different gene expression profiles following long-term exposure to clotrimazole *in vitro*.

**FIG 4:**
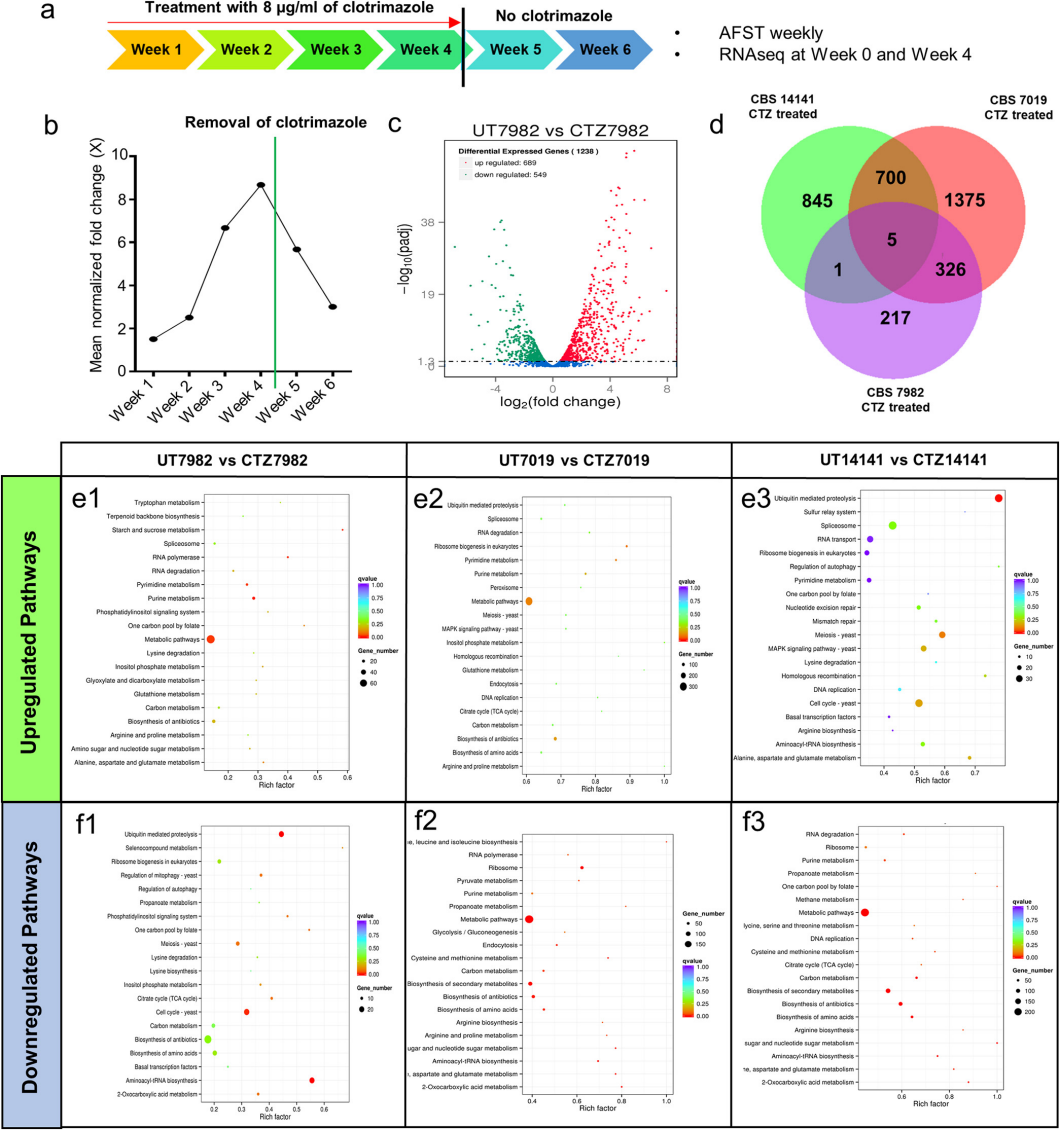
Differentially expressed genes with long-term *in vitro* clotrimazole treatment. (a) Schematic of the 4-week treatment regimen with clotrimazole, followed by removal of clotrimazole after week 4 (black line). (b) Mean normalized fold change of CBS 7982 MICs during 4-week clotrimazole treatment and after clotrimazole removal (green line). (c) Volcano plot of the corresponding upregulated (red) and downregulated (green) genes between untreated CBS 7982 and clotrimazole-treated CBS 7982 at week 4. (d) Venn diagram showing numbers of differentially upregulated exons expressed in the clotrimazole treatment groups. (e and f) Scatterplots for KEGG enrichment showing upregulated (e) and downregulated (f) pathways in untreated (UT) versus clotrimazole treatment (CT) groups.

Long-term (up to 4 weeks) treatment of the wild-type healthy isolate CBS 7982 with clotrimazole (8 μg/ml) (Fig. 4a; Table S3) induced up to an 8-fold increase in clotrimazole MICs (Fig. 4b). After treatment removal at 4 weeks, MICs were observed to fall back to close to starting values. This suggests that elevated MICs may be induced *in vitro* via a transient mechanism. For the strains CBS 7019 and CBS 14141, long-term clotrimazole treatment *in vitro* did not produce any meaningful change in MIC readings, as their starting MICs were already high.

Differential gene expression analysis was performed on RNA-seq of isolates exposed to the above-mentioned MICs of clotrimazole for the three *M. furfur* strains (CBS 7982, CBS 7019 and CBS 14141). Of 1,238 differentially expressed genes between 4-week clotrimazole-treated and nontreated CBS 7982, 689 were upregulated (Fig. 4c and d). Examples of genes commonly upregulated by clotrimazole treatment include *DNM1*, encoding the dynamin protein, and *RBP1P*, encoding the yeast RNA-binding protein, (Table S4), which have been reported to affect antifungal susceptibility and calcineurin-dependent azole tolerance in C. albicans, respectively (30, 31). Metabolic pathways were commonly upregulated in 4-week clotrimazole-treated and nontreated groups for CBS 7982 and CBS 7019 (Fig. 4e1 and e2). KEGG pathways upregulated in clotrimazole-treated CBS 14141 were distinct from those in CBS 7982 and CBS 7019 and included the ubiquitin-mediated proteasome pathway and cell cycle (Fig. 4e3). A reverse trend was observed for KEGG pathways downregulated in the three strains, with metabolic pathways being downregulated in CBS7019 and CBS 14141 and the ubiquitin-mediated proteasome pathway being downregulated in CBS7982 (Fig. 4f1 to f3).

### The ABC transporter encoded by *PDR10* is upregulated after long-term exposure to clotrimazole *in vitro*.

**FIG 5:**
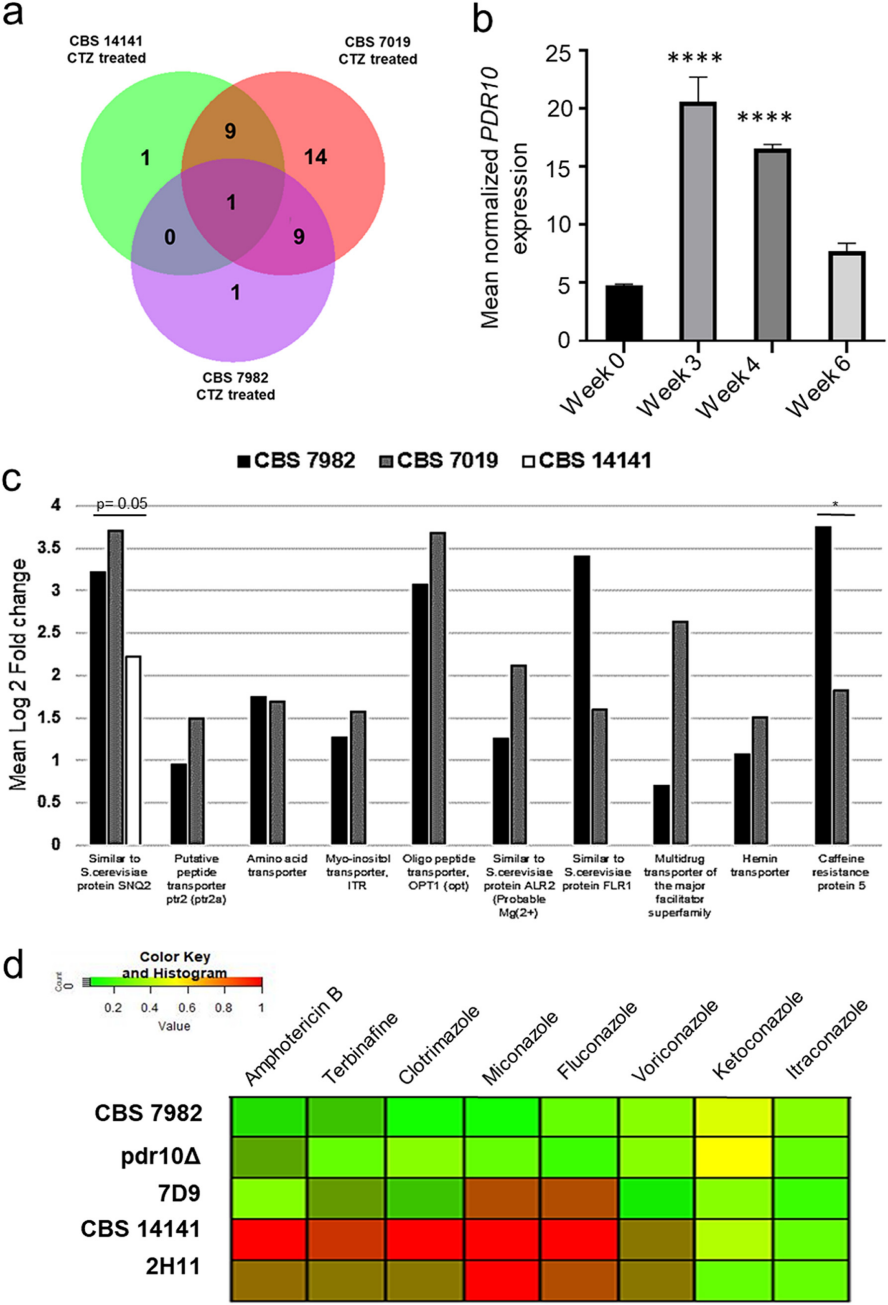
Transporter pump gene expression. (a) Venn diagram showing transmembrane transporter genes which are differentially expressed in clotrimazole-treated CBS 7982, CBS 7019, and CBS 14141. (b) Mean normalized *PDR10* gene expression of CBS 7982 during and after clotrimazole treatment. Values are means and SD. A one-way ANOVA was used to compare each time point against the no-treatment time point, followed by Dunnett’s test (****, *P* < 0.001). (c) Mean log_2_ fold change of key transporter pump genes upregulated after 4-week exposure to clotrimazole in CBS 7982, CBS 7019, and CBS 14141. (d) Heat map of MICs for CBS 7982, CBS 14141, the *pdr10*Δ mutant, and transformants 7D9 and 2H11. MICs were normalized from 0 to 1, with 1 being the highest MIC (red).

Narrowing RNA-seq gene identifications (IDs) to transporter pump proteins identified multiple reads matching exons mapping to “Similar to S. cerevisiae protein, SNQ2.” These reads were consistently the single most highly upregulated transporter gene in all three *M. furfur* strains (Fig. 5). Further, multiple-sequence alignment matched the exon sequence to that of the multidrug transporter encoded by *PDR10*, as described by Ianiri et al. (27). Further analysis of *PDR10* gene expression via reverse transcription-quantitative PCR (RT-qPCR) at baseline and at 3-, 4-, and 6-week time points showed that *PDR10* expression increased over the 4-week clotrimazole treatment period and dropped at 6 weeks, 2 weeks after treatment was withdrawn (Fig. 5b).

RNA-seq analysis also revealed that nine other transporter genes were upregulated in the clotrimazole-treated disease isolates CBS 7019 and CBS 14141 but not CBS 7982, including the previously reported mitochondrial ABC transporter, encoded by *ATM1* (Fig. 5a; Table S5). Other highly upregulated transporter genes in clotrimazole-treated CBS 7982 and CBS 7019 include the oligopeptide protein transporter gene *OPT1*, the multidrug transporter gene *FLR1*, and the caffeine resistance protein gene *CRP5* (Fig. 5c), which have not yet been sequence validated in all isolates of the divergent *M. furfur* strains. These findings suggest that differential expression of transporter genes between different isolates may also affect antifungal susceptibility.

### Deletion of *PDR10* abrogates elevated MICs in disease isolate CBS 14141.

The pleiotropic drug transporter gene *PDR10* was identified to be significantly upregulated in clotrimazole-treated *M. furfur* strain CBS 7982 as described above. It was also found to play a role in *Malassezia* fluconazole resistance by Ianiri et al. (27), for which the CBS 14141 insertional mutants 7D9 and 2H11 and the CRISPR (clustered regularly interspaced palindromic repeats)/Cas9 *pdr10*Δ deletion mutant were constructed using the high-MIC mutant strain CBS 14141. 7D9 has a T-DNA insertion in the putative promoter region of *PDR10*, whereas 2H11 was previously described to have a chromosomal rearrangement mutant involving the gene *ERG5* (27). We confirmed that the *PDR10* gene was absent in the *pdr10*Δ mutant genome and that the *pdr10*Δ mutant showed null *PDR10* expression (Fig. S3).

AFST was performed on the insertional mutants 7D9, 2H11, and the CRISPR/Cas9 *pdr10*Δ mutant alongside wild-type CBS 7982 and mutant CBS 14141 *M. furfur* strains (Fig. 5d; Table 3). Deletion of *PDR10* in CBS 14141 completely reversed the elevated MIC phenotype. The *pdr10*Δ strain showed an AFST profile close to that of the wild type (i.e., CBS 7982) (Fig. 5d). The 7D9 mutant exhibited intermediate susceptibility to the tested azoles, with amphotericin B, terbinafine, and clotrimazole having lower MICs, whereas 2H11 had elevated MICs comparable to those of CBS 14141. Treatment with ABC transport inhibitors (verapamil, carbamazepine, and trifluoperazine) did not reduce antifungal susceptibility in *M. furfur* (data not shown).

**TABLE 3:** MICs for *PDR10* transformants

Strain	MIC (μg/ml) of^*a*^:							
	AMB	TRB	CTZ	MCZ	FLZ	VRZ	KTZ	ITZ
CBS 7982	4	2	4	4	1	0.03	0.03	0.03
PDR10Δ	2	0.25	1	2	2	0.03	0.125	0.125
7D9	1	2	8	32	4	0.5	0.25	0.06
CBS 14141	>32	32	>256	>256	>256	4	0.06	0.06
2H11	4	1	8	>256	128	8	0.25	0.125

a AMB, amphotericin B; TRB, terbinafine; CTZ, clotrimazole; MCZ, miconazole; FLZ, fluconazole; VRZ, voriconazole; KTZ, ketoconazole; ITZ, itraconazole.

These observations are primarily relevant to *M. furfur* strains from the CBS 14141 genomic background (the *pdr10*Δ mutant, 7D9, and 2H11). Analysis of *PDR10* gene expression in other healthy and disease isolates did not show correlation between *PDR10* expression and MIC phenotype (Fig. S3B), suggesting that baseline *PDR10* gene expression may not be an indicator of reduced azole susceptibility in all *M. furfur* strains.

### Wild-type and mutant strains show differences in rhodamine 6G efflux.

**FIG 6:**
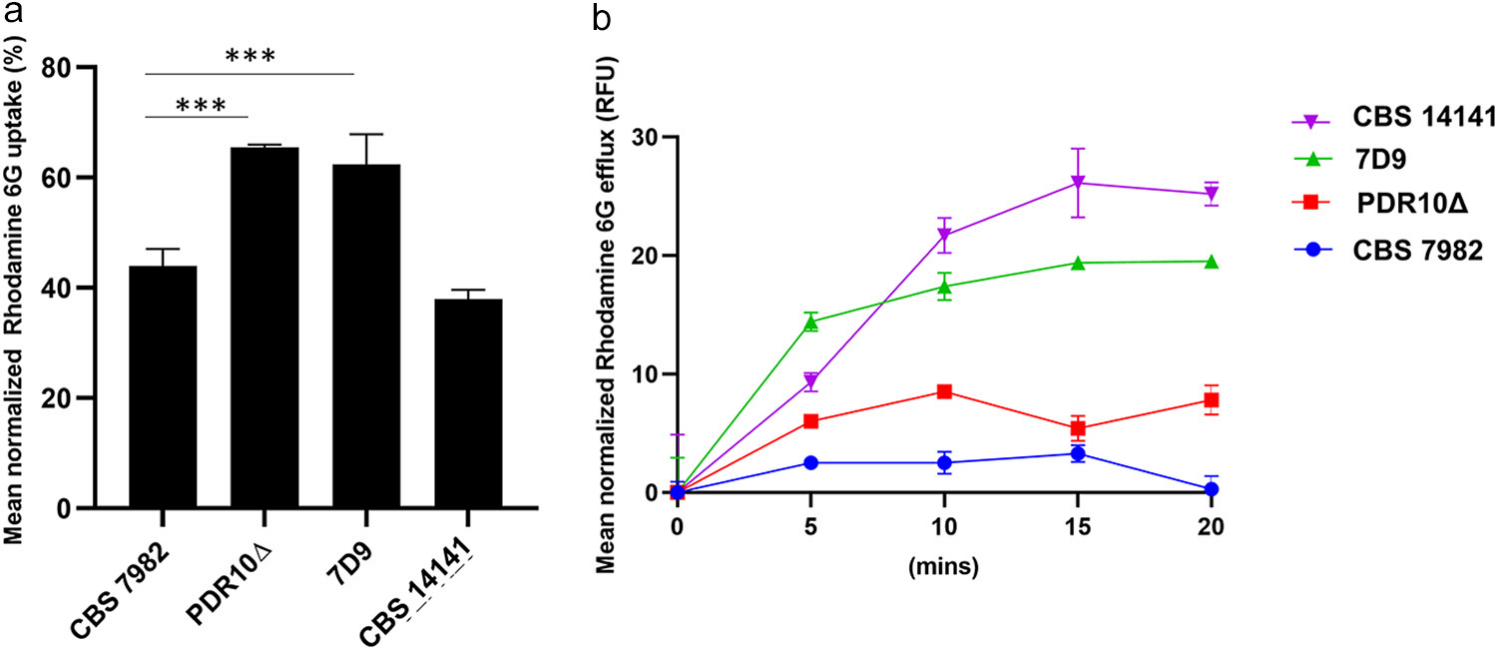
Rhodamine 6G efflux in *PDR10* mutant strains. (a) Percent normalized rhodamine uptake (mean and SD). A one-way ANOVA was used to compare each time point against the no-treatment time point, followed by Dunnett’s test (***, *P* < 0.005). (b) Mean normalized rhodamine 6G efflux (RFU, relative fluorescence units) of wild-type (CBS 7982), mutant (CBS 14141), and transformant (*pdr10*Δ and 7D9) strains over 20 min.

The fluorescent small-molecule dye rhodamine 6G is commonly used to assess the efflux activity of ABC transporters (32). The *pdr10*Δ mutant and 7D9 were observed to have a higher uptake of rhodamine 6G (Fig. 6a) than CBS 7982 and CBS 14141 (Fig. 6a). This is consistent with literature which has reported that rhodamine 6G efflux is correlated with *CDR1* expression (33). CBS 14141 was observed to have the highest rhodamine 6G efflux of the four strains, and CBS 7982 had the lowest (Fig. 6b). Surprisingly, the *pdr10*Δ mutant had rhodamine 6G efflux levels close to those of CBS 7982, suggesting that PDR10 is a key effector of rhodamine 6G efflux. The insertional mutant 7D9 showed intermediate rhodamine 6G efflux levels between those of CBS 7982 and CBS 14141.

## DISCUSSION

In this study, we profiled the antifungal susceptibility of multiple *M. furfur* isolates collected from healthy and disease states and elucidated mechanisms underlying their antifungal susceptibility. Strains isolated from individuals with skin or systemic disease had elevated antifungal MICs relative to strains isolated from healthy individuals. Isolates of *M. furfur* from healthy and disease individuals cluster into groups which reflect their health/disease origin based on their antifungal susceptibility profiles. Isolates from individuals with systemic disease (e.g., blood, urine, and catheter isolates) were the most likely to have elevated MICs. However, exceptions include CBS 9374, which is from a healthy individual but still shows elevated MICs of terbinafine and miconazole. It is likely that the patterns of antifungal susceptibility observed in our disease isolates are stable, given that many of the disease strains have been maintained in culture and passaged repeatedly in fungal banks without antifungal exposure. However, a lack of information on the clinical background of many disease isolates (i.e., antifungal treatment prior to culture isolation and treatment dosage/duration) hampers further interpretation regarding how reduced azole susceptibility could have arisen and whether it is intrinsic or acquired.

Although mutations in *CYP51* are well documented to result in reduced azole susceptibility in fungi (17), this has not been functionally established for *Malassezia*. For *Malassezia*, only *M. globosa* (*MGL_2415*), Malassezia sympodialis (*MSYG_3973*), and *M. pachydermatis* (*Malapachy_4010*) have annotated *CYP51* genes available, with limited literature on the functional role of *CYP51* mutations. Kim et al. described three *CYP51* mutations in an azole-resistant isolate of *M. globosa*, Y127F, A169, and K176N (15), although the extent to which they confer azole resistance was not reported. The large-scale construction of transformants from different strain backgrounds is currently limited by the need for strain-specific sequence data (preferably whole-genome sequencing data) for the design of 1.5-kb flanking arms for homologous recombination required in Agrobacterium tumefaciens-mediated transformation.

The presence of the Y67F mutation in the healthy-scalp isolate 003 SC2 suggests that the mutation alone is insufficient to confer reduced azole susceptibility. A synonymous Y134F mutation in the plant rust fungus Puccinia triticina was documented to have limited impact on its susceptibility to epoxiconazole (34). Our F67Y mutation in CBS 14141 was observed to result in increased amphotericin B susceptibility. This is consistent with observations in Leishmania mexicana, in which a single nucleotide mutation in CYP51 was also reported to induce amphotericin B resistance (35), and suggests a role for this gene in amphotericin B susceptibility.

Rapid transient increases in the mRNA expression of CDR efflux pump genes in C. albicans after exposure to fluconazole have been reported (36, 37). Transporter pump genes such as *PDR10* were observed to be upregulated after exposure to clotrimazole based on RNA-seq data. These genes were validated based on known homology to similar proteins in Saccharomyces cerevisiae or based on existing literature. While the transporter genes *PDR10* (27), *FLR1* (38), and *ITR1* (39) have been reported to be associated with azole resistance, there is little information on the role of other transporter genes, such as *OPT1* and *CRP5*, in multidrug resistance. A putative paralog of *PDR10* was identified immediately adjacent and 3′ to *PDR10* and was identified as *PDR10_2* by Ianiri et al. (27). This warranted further gene validation to ensure that the *pdr10*Δ mutant did not include a knockout of *PDR10_2* (Fig. S3). However, we confirmed that a single knockout of the *PDR10* gene (i.e., *pdr10*Δ) was sufficient to reduce MICs to wild-type levels.

While we demonstrated an increase in clotrimazole MICs after 4 weeks of successive treatment, the effect was dose dependent, and the phenomenon is best observed at antifungal concentrations two to four steps higher than the known MIC for the specific strain. While higher treatment doses may promote higher fold changes in MICs, they are less well tolerated, and the subsequent low viable inoculum impedes susceptibility testing.

At present, *PDR10* expression in CBS 7982 appears to be strongly inducible by the presence of clotrimazole and likely azoles and other stress factors such as chemicals, UV exposure, elevated temperature and nutrient-limiting conditions (27). As described previously, a deletion in *PDR10* for CBS 14141 resulted in increased sensitivity to fluconazole and benomyl, which are antifungal drugs that have different mechanisms of action (27). This suggests a critical role of *PDR10* in nonspecific (or pleiotropic) cellular detoxification in *M. furfur* CBS 14141, most likely through active efflux of xenobiotics, including antifungal drugs, in line with the known function of ABC transporters. Further transcriptional analysis and deletion models in more strains and species of *Malassezia* are required to validate the role of ABC transporters in *Malassezia* azole resistance. This current progress is limited by the poor practical efficiency of generating ATMT plasmid constructs and the need for strain-specific knowledge of genome regions upstream and downstream of the gene of interest. While this has been improved by the development of a CRISPR/Cas9 system (27), challenges remain in the targeting of specific genes (versus unknown homologs) and the role of random insertions as confounding factors.

While rhodamine 6G assays have been useful in giving us an approximation of the relative ABC transporter efflux activity in strains from the same background (i.e., mainly CBS 14141), many transporter pumps are pleiotropic, and more specific binding assays are likely required to narrow down the role of specific pumps across multiple *M. furfur* strains. For the same reasons, the use of generic ABC transport inhibitors, such as verapamil and carbamazepine, was not useful for comparison across different strains.

In summary, we identified differences in azole antifungal susceptibility patterns of 26 isolates of *M. furfur* (13 from healthy subjects and 13 from those with disease). While a Y67F mutation was identified as a contributor to intrinsic resistance in some systemic-disease isolates, its impact on antifungal susceptibility must be interpreted on a strain-by-strain basis in context with other physiological factors. In CBS 7982 and CBS 14141, acquired resistance to clotrimazole and likely other azoles appear to be largely associated with differential activity of ABC transporters, particularly that encoded by *PDR10*. This has been validated by gene expression and functional deletion studies. There are likely additional factors contributing to interstrain differences, which are a result of multiple environmental stresses and other selection pressures. Understanding the functional mechanisms underlying azole resistance in *Malassezia* across a spectrum of strains, sources, and genetic backgrounds will be useful for the identification of new therapeutic targets to prepare for the emergence of new resistant strains.

## MATERIALS AND METHODS

### Strains and culture conditions.

No active primary culture isolation was performed in this study. All 26 isolates used in this study were either obtained from the Westerdijik Fungal Diversity Institute or obtained from previously published studies (40–42). Based on data available in fungal bank records and from previous publications, (13 of the strains were isolated from healthy individuals and 13 from patients with preexisting skin or medical conditions) (Table 1). Strains CBS 14141, JLPK13, Mal18, Mal24, Mal25, Mal26, Mal32, and PM315 were a kind contribution from Bart Theelen and Claudia Cafarchia. All *Malassezia furfur* strains were maintained on modified Dixon agar or broth at 32°C as described previously (10, 40).

### Antifungal susceptibility testing.

Antifungal susceptibility testing was performed using a broth microdilution method as described by Leong et al. (10). Briefly, 200× drug stock dilutions were prepared at a 2× concentration in fresh OptiMAL medium. Amphotericin B, terbinafine, clotrimazole, miconazole, itraconazole, fluconazole, voriconazole, and ketoconazole were purchased from Sigma-Aldrich, Singapore. Stock and drug plate dilutions were prepared in accordance with CLSI and EUCAST guidelines. Yeast inocula were obtained from 4- to 7-day-old strains of *Malassezia* spp. A 50-μl yeast inoculum was added to 50 μl of 2× concentrated antifungals to achieve a final cell density of 5 × 10^3^ to 5 × 10^4^ CFU/ml. A 10-μl portion of 2× yeast inoculum that had been diluted 10 times was also plated onto a modified Leeming-Notman agar plate and incubated for 4 to 7 days at 35°C for postverification of the CFU inoculum (10 to 100 colonies per 10 μl). Each assay was performed in triplicate plates for a single culture at every individual time point or reading.

### Long-term *in vitro* treatment with clotrimazole.

CBS 7982 was maintained in triplicate cultures of mDixon broth containing 8 μg/ml of clotrimazole for 4 weeks at 32°C, with fresh medium and antifungal supplied every alternate day. After 4 weeks, medium was replaced with fresh medium only (i.e., no antifungal) every alternate day for another 2 weeks. A 100-μl portion of culture was removed every 7 days for antifungal susceptibility testing as described above. For CBS 7019 and CBS 14141, clotrimazole concentrations were both 256 μg/ml. RNA extraction was performed on aliquots of each triplicate samples at log phase in week 0 (before addition of clotrimazole) and week 4 (after 4-week clotrimazole treatment) for RNA-seq analysis.

### Gene analysis.

All primer sequences used in this study are listed in Table S5. Unannotated gene sequences for *CYP51* and *PDR10* were derived from a nucleotide BLAST of the whole-genome sequencing (WGS) data (NCBI BioProject database no. PRJNA286710 [43]) or by sequencing the specific gene using the self-designed primers (Table S5). PCR was performed using Platinum *Taq* DNA polymerase (Thermo Fisher, Singapore), TaKaRa HS DNA polymerase (TaKaRa Bio USA Inc.) for 1.5-kb flanking arms, and LongAmp (New England Biolabs [NEB], Singapore). Gel extraction of the PCR product was performed with a QIAquick gel extraction kit per the manufacturer’s instructions (Qiagen, Singapore). Sanger sequencing was performed on the purified PCR product using BigDye (Thermo Fisher, Singapore) per the manufacturer’s instructions. RT-qPCR was performed using the GoTaq one-step RT-qPCR system (Promega, Singapore) or a Superscript transcriptase III kit (Thermo Fisher, Singapore) with the respective gene-specific primers using the *Malassezia* actin gene *ACT1* as the housekeeping gene. PCR with reverse-transcribed cDNA for actin was used to detect genomic-DNA contamination. Relative gene expression was analyzed using the Q-Gene module as described by Muller et al. (44).

Gene sequences derived from Sanger sequencing were translated into protein sequences using the ExPASy translate tool (https://web.expasy.org/translate/) and analyzed using multiple-sequence alignment (http://multalin.toulouse.inra.fr/multalin/multalin.html) (45).

### Agrobacterium tumefaciens-mediated transformation.

For ATMT, 1.5-kb upstream and downstream flanking arms of *CYP51* were derived from PCR (TaKaRa Bio USA Inc.) of CBS 7982 or CBS 14141 genomic DNA and assembled with CBS 7982 *CYP51* gene blocks (TTC/TAC) (Integrated DNA Technologies, Singapore) in the binary vector, pGI3, and the nourseothricin (NAT) and neomycin (NEO) plasmid cassettes pAIM2 and pAIM6 under the control of the *M. sympodialis ACT1* promoter and terminator. Insertional mutagenesis was performed using the A. tumefaciens strain EHA105 as described previously (46).

### RNA-seq.

Total RNA was extracted and purified from log-phase cultures of CBS 7982, CBS 7019, and CBS 14141. Three biological replicates of each sample were analyzed. Briefly, cell pellets were washed three times in sterile phosphate-buffered saline (PBS) before resuspension in TRIzol and frozen at −80°C overnight. On thawing, samples were subjected to bead beating followed by use of the Direct-zol RNA miniprep kit (Zymo Research, USA), following the manufacturer's instructions. An additional DNase step was performed with the Turbo DNase kit (Thermo Fisher, Singapore) per the manufacturer’s instructions. RNA sequencing and differential analysis services were provided by Novogene AIT, Singapore. One microgram of RNA was used for library preparation using a NEBNext Ultra RNA library prep kit for Illumina (NEB, USA) per the manufacturer’s instructions. Next, 125-bp/150-bp paired-end reads were generated from an Illumina-based sequencing platform and processed with the appropriate quality controls. Differential expression analysis (untreated versus clotrimazole treated, healthy versus disease isolate) was performed using the DESeq R package (1.18.0).

### Data and statistical analysis.

All assays were performed as three independent experiments, each with triplicate readings unless otherwise stated. For pairwise comparisons, a paired two-tailed Student's *t* test was performed with Microsoft Excel. For grouped comparisons, a one-way analysis of variance (ANOVA) with Dunnett’s test was performed with GraphPad Prism 8 (GraphPad Software, CA, USA). For heat map plotting of MICs, all MICs were normalized across the different antifungal concentrations such that the highest values were set at 1 and the lowest value were calculated as 1/total number of concentrations. Heat maps and Venn diagrams were plotted using the gplots package in R (version 3.03).

### Rhodamine 6G assay.

The rhodamine 6G efflux assay was performed as described previously (30). Briefly, equal volumes of cells were normalized to an optical density at 600 nm (OD_600_) of 0.2 and incubated with 10 μM rhodamine 6G (Tee Hai Chem, Singapore) in PBS for 30 min at 32°C. Next, cells were transferred to 4°C and spun down to collect the supernatant. Pellets were washed twice with PBS and resuspended in equal volumes of PBS containing 2% glucose and incubated at 32°C. An aliquot of solution was removed at 0-, 5-, 10-, 15-, and 20-min intervals and spun down to collect the supernatant. Each sample was plated in triplicate.

## Supplementary Material

Supplemental file 4

Supplemental file 3

Supplemental file 1

Supplemental file 2

## References

[1] Hyde KD, Al-Hatmi AMS, Andersen B, Boekhout T, Buzina W, Dawson TL, Eastwood DC, Jones EBG, de Hoog S, Kang Y, Longcore JE, McKenzie EHC, Meis JF, Pinson-Gadais L, Rathnayaka AR, Richard-Forget F, Stadler M, Theelen B, Thongbai B, Tsui CKM. 2018. The world’s ten most feared fungi. Fungal Divers 93:161–194. 10.1007/s13225-018-0413-9.

[2] Theelen B, Cafarchia C, Gaitanis G, Bassukas ID, Boekhout T, Dawson TL. 2018. *Malassezia* ecology, pathophysiology, and treatment. Med Mycol 56:S10–S25. 10.1093/mmy/myx134. 29538738

[3] Limon JJ, Tang J, Li D, Wolf AJ, Michelsen KS, Funari V, Gargus M, Nguyen C, Sharma P, Maymi VI, Iliev ID, Skalski JH, Brown J, Landers C, Borneman J, Braun J, Targan SR, McGovern DPB, Underhill DM. 2019. Malassezia is associated with Crohn’s disease and exacerbates colitis in mouse models. Cell Host Microbe 25:377–388.E6. 10.1016/j.chom.2019.01.007. 30850233 PMC6417942

[4] Aykut B, Pushalkar S, Chen R, Li Q, Abengozar R, Kim JI, Shadaloey SA, Wu D, Preiss P, Verma N, Guo Y, Saxena A, Vardhan M, Diskin B, Wang W, Leinwand J, Kurz E, Kochen Rossi JA, Hundeyin M, Zambrinis C, Li X, Saxena D, Miller G. 2019. The fungal mycobiome promotes pancreatic oncogenesis via activation of MBL. Nature 574:264–267. 10.1038/s41586-019-1608-2. 31578522 PMC6858566

[5] Havlickova B, Czaika VA, Friedrich M. 2008. Epidemiological trends in skin mycoses worldwide. Mycoses 51:2–15. 10.1111/j.1439-0507.2008.01606.x. 18783559

[6] Chang HJ, Miller HL, Watkins N, Arduino MJ, Ashford DA, Midgley G, Aguero SM, Pinto-Powell R, von Reyn CF, Edwards W, McNeil MM, Jarvis WR, Pruitt R. 1998. An epidemic of *Malassezia pachydermatis* in an intensive care nursery associated with colonization of health care workers’ pet dogs. N Engl J Med 338:706–711. 10.1056/NEJM199803123381102. 9494146

[7] Chinn R, Pong A, Schultz K, Kim J, Kaegi D, Rasmussen M, Woerle C, Malagon-Maldonado G, Neder C, Beer K, Chow N, Glowicz J, Lockhart S, Jackson B, Litvintseva A. 2017. A cluster of fluconazole-resistant Malassezia pachydermatis in a neonatal intensive care unit—California, 2015–2016. Open Forum Infect Dis 4:S176–S177. 10.1093/ofid/ofx163.321.

[8] Ilahi A, Hadrich I, Goudjil S, Kongolo G, Chazal C, Léké A, Ayadi A, Chouaki T, Ranque S. 2018. Molecular epidemiology of a Malassezia pachydermatis neonatal unit outbreak. Med Mycol 56:69–77. 10.1093/mmy/myx022. 28371911

[9] Archer-Dubon C, Icaza-Chivez ME, Orozco-Topete R, Reyes E, Baez-Martinez R, De León SP. 1999. An epidemic outbreak of Malassezia folliculitis in three adult patients in an intensive care unit: a previously unrecognized nosocomial infection. Int J Dermatol 38:453–456. 10.1046/j.1365-4362.1999.00718.x. 10397586

[10] Leong C, Buttafuoco A, Glatz M, Bosshard PP. 2017. Antifungal susceptibility testing of Malassezia spp. with an optimized colorimetric broth microdilution method. J Clin Microbiol 55:1883–1893. 10.1128/JCM.00338-17. 28381607 PMC5442545

[11] Beardsley J, Halliday CL, Chen SCA, Sorrell TC. 2018. Responding to the emergence of antifungal drug resistance: perspectives from the bench and the bedside. Future Microbiol 13:1175–1191. 10.2217/fmb-2018-0059. 30113223 PMC6190174

[12] Zeina A, Kanafani JRP. 2008. Resistance to antifungal sgents: mechanisms and clinical impact. Clin Infect Dis 46:120–128. 10.1086/524071. 18171227

[13] Rogers PD, Barker KS. 2003. Genome-wide expression profile analysis reveals coordinately regulated genes associated with stepwise acquisition of azole resistance in Candida albicans clinical isolates. Antimicrob Agents Chemother 47:1220–1227. 10.1128/aac.47.4.1220-1227.2003. 12654650 PMC152536

[14] Goldman M, Cloud GA, Smedema M, Lemonte A, Connolly P, Mckinsey DS, Kauffman CA, Moskovitz B, Wheat LJ, Flanigan C, Gutsch H, Weissinger B, Baruch A, Dine A, Smith D, Lee B, Nixon H, Bamberger D, Simpson D, Black J, Norris S, Slama T, Ryan S, Richardson J, McKinsey J, Lancaster D, Ray D, Threlkeld M. 2000. Does long-term itraconazole prophylaxis result in in vitro azole resistance in mucosal Candida albicans isolates from persons with advanced human immunodeficiency virus infection? Antimicrob Agents Chemother 44:1585–1587. 10.1128/aac.44.6.1585-1587.2000. 10817713 PMC89917

[15] Kim D, Lim YR, Ohk SO, Kim BJ, Chun YJ. 2011. Functional expression and characterization of CYP51 from dandruff-causing Malassezia globosa. FEMS Yeast Res 11:80–87. 10.1111/j.1567-1364.2010.00692.x. 21114623

[16] Park M, Cho YJ, Lee YW, Jung WH. 2020. Genomic multiplication and drug efflux influence ketoconazole resistance in Malassezia restricta. Front Cell Infect Microbiol 10:191. 10.3389/fcimb.2020.00191. 32426297 PMC7203472

[17] Parker JE, Warrilow AGS, Price CL, Mullins JGL, Kelly DE, Kelly SL. 2014. Resistance to antifungals that target CYP51. J Chem Biol 7:143–161. 10.1007/s12154-014-0121-1. 25320648 PMC4182338

[18] Kim M, Cho YJ, Park M, Choi Y, Hwang SY, Jung WH. 2018. Genomic tandem quadruplication is associated with ketoconazole resistance in malassezia pachydermatis. J Microbiol Biotechnol 28:1937–1945. 10.4014/jmb.1810.10019. 30562885

[19] Warrilow AGS, Price CL, Parker JE, Rolley NJ, Smyrniotis CJ, Hughes DD, Thoss V, Nes WD, Kelly DE, Holman TR, Kelly SL. 2016. Azole antifungal sensitivity of sterol 14α-demethylase (CYP51) and CYP5218 from malassezia globosa. Sci Rep 6:27690. 10.1038/srep27690. 27291783 PMC4904373

[20] Angileri M, Pasquetti M, De Lucia M, Peano A. 2019. Azole resistance of Malassezia pachydermatis causing treatment failure in a dog. Med Mycol Case Rep 23:23–58. 10.1016/j.mmcr.2018.12.004. 30662826 PMC6325069

[21] Kano R, Yokoi S, Kariya N, Oshimo K, Kamata H. 2019. Multi-azole-resistant strain of Malassezia pachydermatis isolated from a canine Malassezia dermatitis. Med Mycol 57:346–350. 10.1093/mmy/myy035. 29800467

[22] Prasad R, Rawal MK. 2014. Efflux pump proteins in antifungal resistance. Front Pharmacol 5:202. 10.3389/fphar.2014.00202. 25221515 PMC4148622

[23] Wasi M, Kumar Khandelwal N, Moorhouse AJ, Nair R, Vishwakarma P, Bravo Ruiz G, Ross ZK, Lorenz A, Rudramurthy SM, Chakrabarti A, Lynn AM, Mondal AK, Gow NAR, Prasad R. 2019. ABC transporter genes show upregulated expression in drug-resistant clinical isolates of candida auris: a genome-wide characterization of atp-binding cassette (abc) transporter genes. Front Microbiol 10:1445. 10.3389/fmicb.2019.01445. 31379756 PMC6647914

[24] Costa C, Dias PJ, Sá-Correia I, Teixeira MC. 2014. MFS multidrug transporters in pathogenic fungi: do they have real clinical impact? Front Physiol 5:197. 10.3389/fphys.2014.00197. 24904431 PMC4035561

[25] Choi MJ, Won EJ, Shin JH, Kim SH, Lee WG, Kim MN, Lee K, Shin MG, Suh SP, Ryang DW, Im YJ. 2016. Resistance mechanisms and clinical features of fluconazole-nonsusceptible Candida tropicalis isolates compared with fluconazole-less-susceptible isolates. Antimicrob Agents Chemother 60:3653–3661. 10.1128/AAC.02652-15. 27044550 PMC4879413

[26] Cafarchia C, Iatta R, Immediato D, Puttilli MR, Otranto D. 2015. Azole susceptibility of Malassezia pachydermatis and Malassezia furfur and tentative epidemiological cut-off values. Med Mycol 53:743–748. 10.1093/mmy/myv049. 26162472

[27] Ianiri G, Dagotto G, Sun S, Heitman J. 2019. Advancing functional genetics through Agrobacterium-mediated insertional mutagenesis and CRISPR/Cas9 in the commensal and pathogenic yeast Malassezia. Genetics 212:1163–1179. 10.1534/genetics.119.302329. 31243056 PMC6707463

[28] Chen CK, Leung SSF, Guilbert C, Jacobson MP, Mckerrow JH, Podust LM. 2010. Structural characterization of CYP51 from Trypanosoma cruzi and Trypanosoma brucei bound to the antifungal drugs posaconazole and fluconazole. PLoS Negl Trop Dis 4:e651. 10.1371/journal.pntd.0000651. 20386598 PMC2850312

[29] Warrilow AG, Nishimoto AT, Parker JE, Price CL, Flowers SA, Kelly DE, David Rogers P, Kelly SL. 2019. The evolution of Azole resistance in Candida albicans Sterol 14-demethylase (CYP51) through incremental amino acid substitutions. Antimicrob Agents Chemother 63:e02586-18. 10.1128/AAC.02586-18. 30783005 PMC6496074

[30] Truong T, Zeng G, Lim TK, Cao T, Pang LM, Lee YM, Lin Q, Wang Y, Seneviratne CJ. 2020. Proteomics analysis of Candida albicans dnm1 haploid mutant unraveled the association between mitochondrial fission and antifungal susceptibility. Proteomics 20:1900240. 10.1002/pmic.201900240. 31811746

[31] Cannon RD, Lamping E, Holmes AR, Niimi K, Tanabe K, Niimi M, Monk BC. 2007. Candida albicans drug resistance—another way to cope with stress. Microbiology (Reading) 153:3211–3217. 10.1099/mic.0.2007/010405-0. 17906120

[32] Bhattacharya S, Sobel JD, White TC. 2016. A combination fluorescence assay demonstrates increased efflux pump activity as a resistance mechanism in azole-resistant vaginal Candida albicans isolates. Antimicrob Agents Chemother 60:5858–5866. 10.1128/AAC.01252-16. 27431223 PMC5038269

[33] Choi CH. 2005. ABC transporters as multidrug resistance mechanisms and the development of chemosensitizers for their reversal. Cancer Cell Int 5:30. 10.1186/1475-2867-5-30. 16202168 PMC1277830

[34] Stammler G, Cordero J, Koch A, Semar M, Schlehuber S. 2009. Role of the Y134F mutation in cyp51 and overexpression of cyp51 in the sensitivity response of Puccinia triticina to epoxiconazole. Crop Prot 28:891–897. 10.1016/j.cropro.2009.05.007.

[35] Mwenechanya R, Kovářová J, Dickens NJ, Manikhandan M, Herzyk P, Vincent IM, Weidt SK, Burgess KE, Burchmore RJS, Pountain AW, Smith TK, Creek DJ, Kim DH, Lepesheva GI, Barrett MP. 2017. Sterol 14α-demethylase mutation leads to amphotericin B resistance in Leishmania mexicana. PLoS Negl Trop Dis 11:e0005649. 10.1371/journal.pntd.0005649. 28622334 PMC5498063

[36] Marr KA, Lyons CN, Rustad T, Bowden RA, White TC. 1998. Rapid, transient fluconazole resistance in Candida albicans is associated with increased mRNA levels of CDR. Antimicrob Agents Chemother 42:2584–2589. 10.1128/AAC.42.10.2584. 9756759 PMC105901

[37] Marr KA, Lyons CN, Ha K, Rustad TR, White TC. 2001. Inducible azole resistance associated with a heterogeneous phenotype in Candida albicans. Antimicrob Agents Chemother 45:52–59. 10.1128/AAC.45.1.52-59.2001. 11120944 PMC90239

[38] Brôco N, Tenreiro S, Viegas CA, Sá-Correia I. 1999. FLR1 gene (ORF YBR008c) is required for benomyl and methotrexate resistance inSaccharomyces cerevisiae and its benomyl-induced expression is dependent on Pdr3 transcriptional regulator. Yeast 15:1595–1608. 10.1002/(SICI)1097-0061(199911)15:15<1595::AID-YEA484>3.0.CO;2-6. 10572257

[39] Claus S, Jezierska S, Van Bogaert INA. 2019. Protein-facilitated transport of hydrophobic molecules across the yeast plasma membrane. FEBS Lett 593:1508–1527. 10.1002/1873-3468.13469. 31166012

[40] Leong C, Schmid B, Toi MJ, Wang J, Irudayaswamy AS, Goh JPZ, Bosshard PP, Glatz M, Dawson TL. 2019. Geographical and ethnic differences influence culturable commensal yeast diversity on healthy skin. Front Microbiol 10:1891. 10.3389/fmicb.2019.01891. 31551938 PMC6736582

[41] Boekhout T, Kamp M, Guého E. 1998. Molecular typing of Malassezia species with PFGE and RAPD. Med Mycol 36:365–372. 10.1080/02681219880000581. 10206745

[42] Iatta R, Figueredo LA, Montagna MT, Otranto D, Cafarchia C. 2014. In vitro antifungal susceptibility of Malassezia furfur from bloodstream infections. J Med Microbiol 63:1467–1473. 10.1099/jmm.0.078709-0. 25168965

[43] Wu G, Zhao H, Li C, Rajapakse MP, Wong WC, Xu J, Saunders CW, Reeder NL, Reilman RA, Scheynius A, Sun S, Billmyre BR, Li W, Averette AF, Mieczkowski P, Heitman J, Theelen B, Schröder MS, De Sessions PF, Butler G, Maurer-Stroh S, Boekhout T, Nagarajan N, Dawson TL. 2015. Genus-wide comparative genomics of Malassezia delineates its phylogeny, physiology, and niche adaptation on human skin. PLoS Genet 11:e1005614. 10.1371/journal.pgen.1005614. 26539826 PMC4634964

[44] Muller PY, Janovjak H, Miserez AR, Dobbie Z. 2002. Processing of gene expression data generated by quantitative real-time RT-PCR. Biotechniques 32:1372–1374, 1376, 1378–1379. 12074169

[45] Corpet F. 1988. Multiple sequence alignment with hierarchical clustering. Nucleic Acids Res 16:10881–10890. 10.1093/nar/16.22.10881. 2849754 PMC338945

[46] Ianiri G, Averette AF, Kingsbury JM, Heitman J, Idnurm A. 2016. Gene function analysis in the ubiquitous human commensal and pathogen Malassezia genus. mBio 7:e01853-16. 10.1128/mBio.01853-16. 27899504 PMC5137500

