# Supplementary material for: Azole Resistance Mechanisms in Pathogenic Malassezia furfur
Source: Antimicrob Agents Chemother. 2021 Apr 19;65(5):e01975-20. doi: 10.1128/AAC.01975-20 (PMC8092866; doi:10.1128/AAC.01975-20)
Supplement: Supplemental file 1 [file AAC.01975-20-s0001.pdf]

Supplementary Material

Azole resistance mechanisms in pathogenic *M. furfur*

Cheryl Leong[1], Joel Chan Wai Kit[1], Shi Mun Lee[1], Yuen In Lam[1], Joleen P.Z. Goh[1], Giuseppe Ianiri[2], and Thomas L. Dawson, Jr[1,3#]

[1]Skin Research Institute of Singapore, Agency for Science, Technology and Research (A*STAR), Singapore, Singapore,

[22]Department of Agricultural, Environmental and Food Sciences, University of Molise, 86100, Campobasso, Italy

[3]Center for Cell Death, Injury & Regeneration, Departments of Drug Discovery & Biomedical Sciences and Biochemistry & Molecular Biology, Medical University of South Carolina, Charleston, SC

#Address correspondence to:

Thomas Dawson

Skin Research Institute of Singapore

11 Mandalay Road, #17-01,

Singapore 308232

Supplementary Figures

Supplementary Figure 1. Conserved motifs in *M. furfur* CYP51

Supplementary Figure 2. Y67F mutation at nucleotide level

Supplementary Figure 3. Validation of *PDR10* gene KO

Supplementary Tables

Supplementary Table 1. MIC values for 26 *M. furfur* isolates

Supplementary Table 2. Top 20 upregulated genes in comparison of untreated CBS 7982, CBS 7019 and CBS 14141- Excel sheet

Supplementary Table 3. MIC values for CBS 7982 over six weeks

Supplementary Table 4. Genes upregulated after clotrimazole treatment – Excel sheet

Supplementary Table 5. Transporter genes upregulated after clotrimazole treatment - Excel sheet

Supplementary Table 6. Primer sequences

Supplementary Figure 1:

Conserved regions of *CYP51A1 M. globosa* (reference sequence) versus *M. furfur*

Key conserved *CYP51* gene regions identified in M. globosa[1]

- Putative heme-binding site (^{471}FGAGRHRCIG^{480})
- EXXR motif (^{365}ERLR^{368})
- Conserved threonine of I-helix involved in proton delivery (^{307}T)

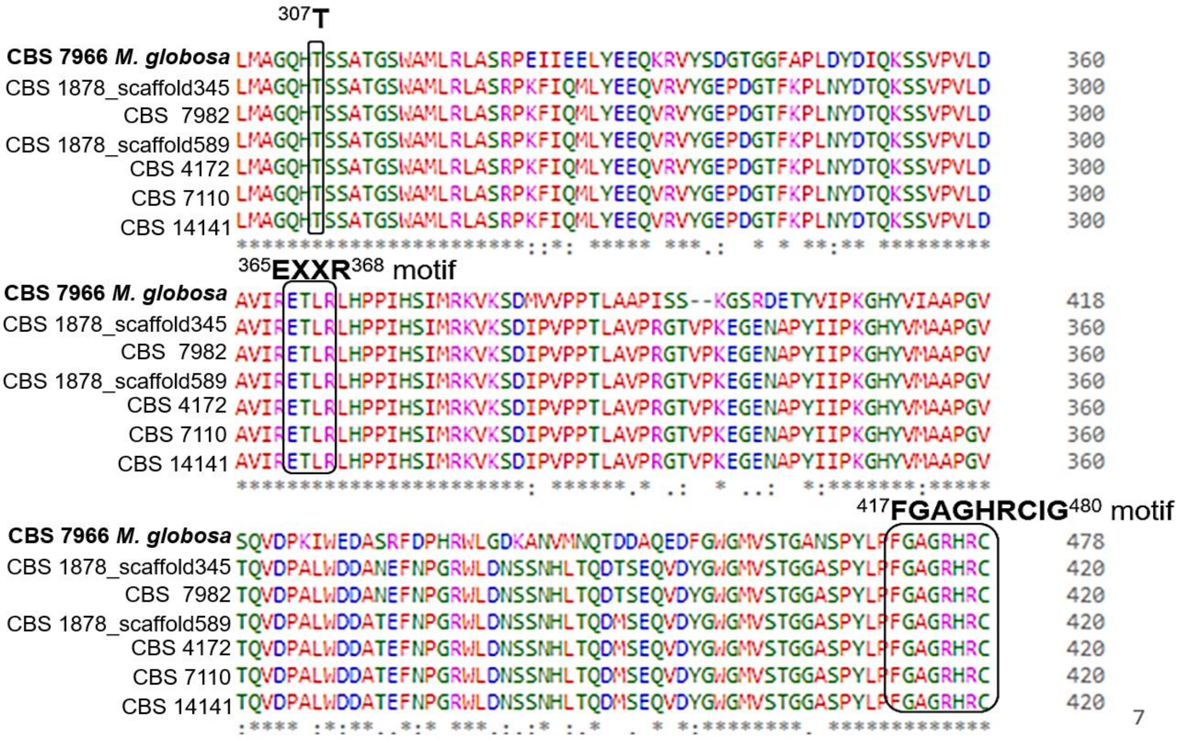

Supplementary Figure 2.

Y67F mutation at nucleotide level: Strains with A to T mutation are highlighted in yellow

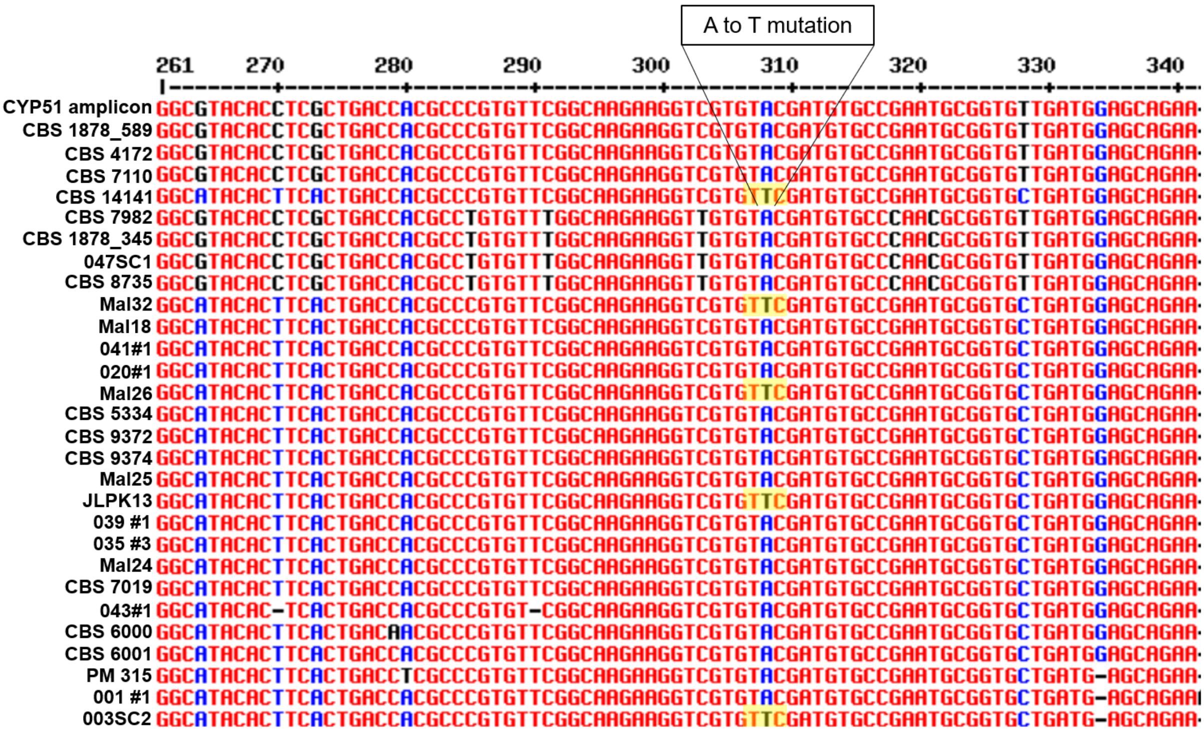

Supplementary Figure 3.

Validation of *PDR10* gene knockout and gene expression in *M. furfur* strains

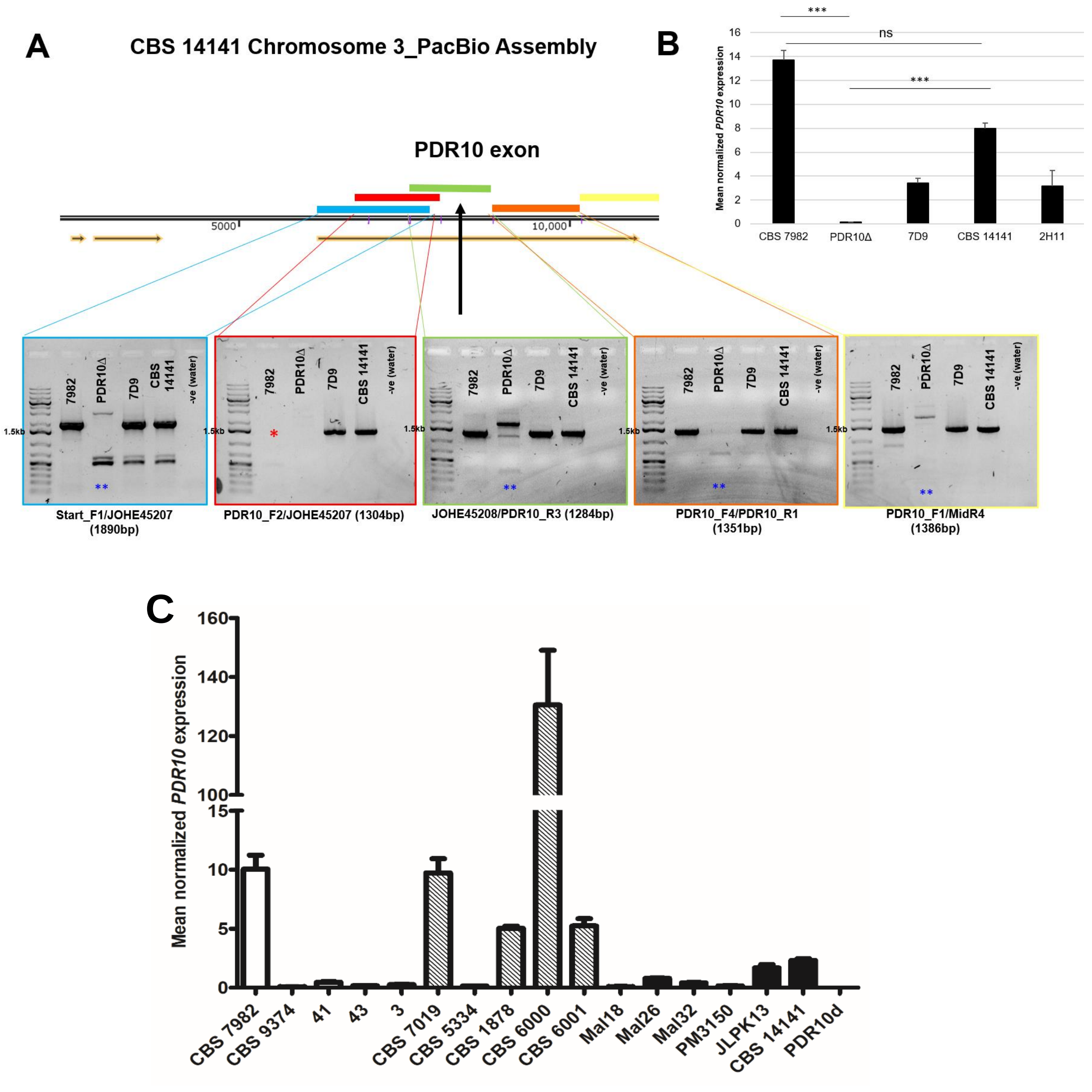

Supplementary Table 1. MIC values for 26 *M. furfur* isolates

		Minimum Inhibitory Concentrations (MICs)/ µg/ml							
**Strain**	**Source**	**AMB/(GM)**	**TRB/(GM)**	**CTZ/(GM)**	**MCZ/(GM)**	**ITZ/(GM)**	**FLZ/(GM)**	**KTZ/(GM)**	**VRZ/(GM)**
**Disease Isolates**									
**CBS 14141**	Blood	>32	8-32 (16)	>256	>256	0.03-0.06 (0.04)	>256	0.125	4
**CBS6000**	Dandruff	1-4 (1.74)	8-16 (10.1)	16-128 (76.1)	256	0.06	8	1	0.125
**CBS6001**	Pityriasis versicolor	2-4 (2.82)	8-16 (11.3)	8-16 (12.7)	16	0.06	8	1	0.125
**JLPK13**	Urine	2-16 (4.42)	16->32 (26.9)	256	256	0.06	64-128 (76.1)	0.25-0.5	1-2 (1.59)
**CBS5334**	Infected Skin	0.25-1 (0.44)	16	16-128 (40.3)	64-256 (128)	0.03-0.06 (0.047)	4-8 (6.34)	0.25-1 (0.40)	0.125
**Mal 18**	Blood	1-4 (2)	4-8 (6.35)	32-64 (50.8)	64	0.03-0.06 (0.038)	4	0.25-0.5 (0.40)	0.125
**Mal 24**	Blood	1-4 (2)	2	32	32-64 (45.3)	0.03	4	0.25-1 (0.5)	0.06-0.125 (0.077)
**Mal 25**	Skin catheter insertion	1	4-16 (8)	64-256 (128)	256	0.03-0.125 (0.077)	4-8 (6.35)	0.5-1 (0.63)	0.125
**Mal 26**	Blood	2-16 (6.72)	8-16 (12.1)	>256	128->256	0.03-0.125 (0.078)	128	0.5-2 (1)	1-4 (1.59)
**Mal 32**	Skin catheter insertion	16	>32	>256	128->256	0.125	64-128 (101.6)	0.5-2 (1)	1-2 (1.59)
**PM 315**	Anal Swab of Neonate	1-4 (2)	8-32 (20.2)	128->256	128->256	0.06	8	0.5-2 (0.79)	0.25-0.5 (0.31)
**CBS 1878**	Dandruff	1-2 (1.41)	16->32 (25.4)	128-256 (203.2)	64-256 (128)	0.06	4	1-4 (2)	0.125
**CBS 7019**	Pityriasis Versicolor	1-4 (1.59)	8-16 (10.1)	128-256 (161.3)	256	0.03	8-16 (10.1)	0.5-1 (0.63)	0.125

GM: Geometric mean of n ≥ 3 replicates – value not indicated if all MIC values are equal across all replicates

AMB: Amphotericin B
CTZ: Clotrimazole
ITZ: Itraconazole
KTZ; Ketoconazole
TRB: Terbinafine
MCZ: Miconazole
FLZ: Fluconazole
VRZ: Voriconazole

		Minimum Inhibitory Concentrations (MICs)/ µg/ml							
**Strain**	**Source**	**AMB/(GM)**	**TRB/(GM)**	**CTZ/(GM)**	**MCZ/(GM)**	**ITZ/(GM)**	**FLZ/(GM)**	**KTZ/(GM)**	**VRZ/(GM)**
**Healthy Isolates**									
**CBS 7982**	Healthy Skin, ear	1-2 (1.23)	0.25-0.5 (0.4)	1-8 (3.17)	0.5-4 (1.59)	0.03	1	≤0.03	0.03
**020#01**	Healthy Skin, nose	1-4 (2.52)	1-2 (1.41)	2-4	4	0.06	4	0.06	0.125
**041#01**	Healthy Skin, nose	4	4	16	16	0.06	4	≤0.03	0.125
**039#01**	Healthy Skin, nose	1-4 (2)	2-8 (4)	8-16(10.1)	8->16	0.06	2	≤0.03	0.06
**035#03**	Healthy Skin, nose	2-8 (4)	2-4 (3.17)	8-16 (10.1)	8-16 (10.1)	0.06-0.25 (0.12)	2-4 (2.83)	≤0.03	0.06
**CBS 9372**	Back of healthy individua	1-4 (2)	4-8 (4)	64	128-256 (181.0)	0.03-0.06 (0.038)	2	0.25	0.06
**CBS 9374**	Chest of healthy individual	2	4-8 (5.66)	64-256 (90.5)	64-256 (128)	0.06-0.25 (0.155)	2-8 (3.17)	0.25-2 (0.63)	0.06-0.5 (0.15)
**CBS 8735**	Bronchial wash	16	4-16 (8)	1	1	≤0.03	2-4 (2.52)	≤0.03	0.06-0.125 (0.077)
**047 SC1**	Healthy Scalp isolate	4	2	0.5	2	0.125	2-4 (2.83)	≤0.03	0.125
**003 SC2**	Healthy Scalp isolate	4	2	16	16-64 (40.3)	0.03-0.06 (0.048)	32	≤0.03	0.5
**CBS 7710**	Skin of man	16	2	4	2	0.25-0.5 (0.35)	8	≤0.03	0.125-0.25(0.16)
**043#01**	Healthy Skin, nose	4	8-16 (11.3)	16	8-16 (11.3)	0.06	4	0.03	0.125
**001#01**	Healthy Skin, nose	2	16	128	64	0.125	4	0.5	0.125

GM: Geometric mean of n ≥ 3 replicates – value not indicated if all MIC values are equal across all replicates

AMB: Amphotericin B
TRB: Terbinafine
CTZ: Clotrimazole
MCZ: Miconazole
ITZ: Itraconazole
FLZ: Fluconazole
KTZ; Ketoconazole
VRZ: Voriconazole

Supplementary Table 3.

	Clotrimazole (µg/ml) MIC range[1]	Mean MIC (µg/ml)
**Week 0**	1-4	2
**Week 1**	1-4	3
**Week 2**	2-8	5
**Week 3**	4-16	13.33
**Week 4**	4-32	17.33
**Week 5**	2-16	11.33
**Week 6**	1-4	6

Supplementary Table 6.

Primers (10 µM)	Forward (5'-3')/Reverse (5'-3')
CYP51 Y67F region	CGTGCTTTGGCAGTTGCT/AGAACTTGCTCATCAGGC
7982_F1 1.5kb forward flanking arm	GTTTTCCCAGTCACGACGTTGTAAAACCGTATATCTCACACAGATCG/ CGAGGATCTGCACCGTGGA CCGTAGGTCACCGCCGAGC
7982_F2 1.5kb reverse flanking arm	CTGGGTCAGACATAGGAGAGGACG GCGGCGATTCTTTTCCACCC/ CAGCTATGACATGATTACGAATTCTTAATT CAGCGAGTTGTAGAAGAACC
14141_F1	CTGCAAGGCGATTAAGTTG GGAGGCACATCTATATGGCTTTCC/ CGAGGATCTGCACCGTGGA ACCGCCGAGCCCACTA
14141_F2	CTGGGTCAGACATAGGAGAGGACG AGAAGCCTAGTGTTGTGGTCTAC /CAGCTATGACATGATTACGAATTCTTAATT GGCTGTAGTTGTGTGCCCA
ITS primer (5F4R)	TCCGTAGGTGAACCTGCGG/ TCCTCCGCTTATTGATATGC
Actin	CCCGCTGAACCCSAAGGC/ CACACCGTCACCCGAGTC
PDR10	ATGTTCCTGGCGTACTACCG/ GACAGCACGAGCGACATAAA

[1] Values represent a range observed over triplicate AFST assays performed at each time point
